# Dr. Bennett Dowler

**Published:** 1851-10

**Authors:** 


*Dr. Bennett Dowler.* — Dr. Dowler, of New Orleans, author of numerous scientific papers containing original and ingenious experimental observations on various physiological subjects, has been appointed to the Chair of Physiology and Pathological Anatomy, in the Memphis (Tenn.) Medical College. This institution has lately been reorganized, and a lecture session is announced for the comming winter.

